# Hospital Meetings, &c.

**Published:** 1899-03-11

**Authors:** 


### The Institutional Workshop.

### HOSPITAL MEETINGS, &C.

### THE LEAGUE OF MERCY.

In the report of the meeting of the League of Mercy held on Wednesday (the 1st inst.), at Marlborough House, the Prince of Wales, through an error in transcription, is reported to have said, "I hope you will also observe that the Order is to be *for* one Class only." This should have been "I hope you will a'so observe that the Order is to be one Class only."

### LONDON HOSPITAL.

A quarterly general Court of the Governors of the London Hospital was held on Wednesday, March 1st, Mr. John Hampton Hale presiding. The report giving a record of the work done during the past year was presented and adopted *nem. con.* It states that in 1898 there were treated in the hospital 178,833 out-patients, 11,622 in-patients, and 2,592 children under twelve years of age. These figures show an increase of 17,805 out-patients, 476 in-patients, and 182 children upon the numbers of 1897. The pressure both in the wards and in the out-patient department has at times been extremely severe, but it has always been met cheerfully and successfully by the staff—medical, surgical, and nursing. The audit of the accounts is not yet complete, but the following is an approximate abstract of the items quoted. The total ordinary income was £45,000; the extraordinary income £87,000; the ordinary expenditure was £66,000; the extraordinary £27,000. Of this £12,500 is in reality part of that capital expenditure of more than £100,000 to which the hospital, by accepting the annual grant of £5,000 from the Prince of Wales's Fund is pledged. There has been a serious falling off in the amount received as legacies. Over the last seven years the average has been £18,000. In 1898 not more than £10,000 was received. There has been a balance of about £40,000 to invest temporarily till needed for the buildings, and the committee have bought the following stock: Bank of England, £7,000; Canada Three per Cents., £5,000; and Nottingham Joint Station Three per Cents., £15,000. At the first meeting of the House Committee the Hon. Sydney Holland was unanimously re-elected chairman of the hospital. The committee report progress in the scheme of improvements and extension which has been sanctioned. The Bill to enable the hospital to obtain sites for a new out-patient department and isolation block or nurses' house is now before Parliament, and has been read a first time. The scheme for the improvement of the Romford Street property has been approved by the London County Council, and it is hoped that the Whitechapel Vestry will also grant its consent, so that the work may now be begun with as little delay as possible. It is also hoped that the magistrates will allow Charlotte Court to be closed. Lord Rowton can then begin one of his model dwellings for workmen on the site. It is hardly necessary to point out that when these schemes are carried out not only will the value of the property held by the hospital in the neighbourhood be increased, but the tenants of the hospital, necessarily disturbed during the work, will have greatly improved dwelling accommodation provided for them. The temporary wards are now almost ready for the reception of the one hundred patients for whom there are beds. A further small addition will, if possible, be made, which will add greatly to the comfort of the patients and allow the wards to be more easily worked. The temporary electric and photographic departments are complete and in full working order. It would be difficult to overestimate the value to the successful treatment of disease and accidents of the extension and development of this branch. The ventilation of the out-patients' department is now being proceeded with. The committee hesitated to incur the expense of putting up their own electric light installation, and have decided to see whether satisfactory arrangements can be made with the manager of the proposed electric light station of the Whitechapel district whence it would be possible to obtain a supply of electricity for the lighting of the hospital and other purposes. To keep up the supply of nurses who had received preliminary training which has been found so valuable, the committee have decided to increase the accommodation of Tredegar House. Negotiations have therefore been entered into with Lord Tredegar to take a lease of the adjoining house, 97, Bow Road, and throw the two into one. On the private nursing staff the numbers have risen to 99. As recently 97 of these nurses have been out in attendance upon private cases at the same time the hospital may look for a considerable increase in the amount which the private nursing institution annually contributes to its funds. In connection with the election of Mr. Hora, of 123, Victoria Street, as a vice-president, in recognition of his gift to the Samaritan Society of the London Hospital, the committee hope to obtain increased support for this society. Many patients are discharged from the wards cured indeed of their complaints, but not really sufficiently recovered in strength to return to work. To such, a week or a fortnight at a convalescent home would make all the difference, nor would there be the same likelihood of their breaking down again and having to be readmitted to the wards. Efforts are being made to obtain a convalescent home to be entirely devoted to patients from this hospital suffering from open wounds, and it is much to be hoped that Mrs. Spender and Miss Gully will receive from the Press and from the public the support their generous scheme deserves. Among the donations to the hospital the committee report an anonymous gift of £22,000, to be devoted to the purchase of a site and the building of an isolation block. Seven hundred and fifty feather pillows were presented to the hospital by the *Sun* newspaper; this number has been further increased by friends, so that now every ward is fully supplied, the comfort of the patients being greatly enhanced thereby. Mr. Treves, on his retirement, has been elected Emeritus Professor of Surgery, and has promised to deliver a course of lectures annually in the medical college. The committee announce with regret the serious illness of the house governor and secretary, Mr. G. Q. Roberts, who is now away on sick leave in Madeira, and hopes soon to be sufficiently recovered to resume work at the hospital.

After the adoption of the report the court was made special, and Mr. Hora was elected a vice-president and sanction given to an application to the Tower Hamlets Justices to close Charlotte Court. Several leases were sealed, among them one to Messrs. Charrington and Co. (Limited) of the Anchor beerhouse, Turner Street, for a term of forty years at a rent of £26 10s. per annum. In connection with this last, a telegram was sent from the London United Temperance Council to the chairman, on behalf of the temperance supporters of the London Hospital, protesting against the proposal, but no notice was taken of this, criticism being confined to the question of the adequacy of the price obtained. The Chairman promised that information on this point should be afforded at the next meeting, and a vote of thanks closed the proceedings.

### ROYAL LONDON OPHTHALMIC HOSPITAL.

On Friday last, 3rd inst., Mr. H. P. Sturgis presided at the annual general meeting of the Royal London Ophthalmic Hospital, Moorfields. From the accounts presented it appeared that the ordinary income for 1898 was £4,679, and the extraordinary income—made up of £2,888 in legacies and £450 from the Prince of Wales's Fund—£3,338; together making a total income of £8,018. The ordinary expenditure was £8,055, leaving a debit balance on the year of £37. The in-patients numbered 2,227, and the out-patients 25,713, whose attendances numbered 101,543. Before the end of the present year—in fact, early this summer, it is hoped—the new premises in the City Road will be opened and in occupation. They are being equipped according to modern ideas, and the committee express the hope that they will be found worthy the name and fame of the hospital. The inquiry officer appointed some years since to prevent abuses of the charity, was obliged during the past year to refuse no less than 528 applicants, who, in nearly every case, admitted that they were able to pay a surgeon, but said they were not aware that the hospital was open for the poor only. The report and accounts were adopted *nem. con.* The retiring members of the committee were re-elected, and votes of thanks to the officers and staff, to the Press for aid by publicity, and to the chairman for presiding, brought the proceedings to a close.

### CITY OF LONDON HOSPITAL FOR DISEASES OF THE CHEST, VICTORIA PARK.

The customary placidity of hospital gatherings was considerably varied on the occasion of the annual general meeting of the City of London Hospital for Diseases of the Chest, Victoria Park, held at Gresham College, in the City, on Monday, 6th instant. The proceedings throughout were followed with the keenest interest by the large number of working-men governors present, and for two hours and a half the chief subject of interest to them—the question of the inquiry officer and his inquisition—was kept constantly on the carpet in one shape or another. Every reference in the remotest degree related to the matter was punctuated by aggressive cheers or cries of dissent in such volume as to prove the presence of an overwhelming majority determinedly opposed to the innovation. So obvious was the sense of the meeting that the chairman was clearly justified in the tactics he adopted, in the interest of other sections of the governing body, of postponing any decisive vote, and so giving them an opportunity of being better represented before definite steps, which might have far-reaching consequences, were taken.

The Chairman of the Committee of Management (Mr. Joseph Benson, L.C.C.) presided, and moved the adoption of the report and accounts. From these and from the chairman's remarks in proposing the resolution it appeared that the institution was in a much more satisfactory and hopeful state than had not long since been the case. Last year, out of a total of 164 beds, only 40 were open; now they had 126, of which 119 were actually occupied. They had effected economies in the working, and while the hospital had never been in better condition, and the interests of the patients had not suffered in the least, yet they had reduced their expenses by £3,000 since 1895. Last year they owed about £3,000, while this year they had paid everybody and had also replaced the small investment of £1,000 which constituted their reserve. The ordinary income for 1898 amounted to £11,928, and the ordinary expenditure to £8,637. The annual subscriptions were £2,664, an increase of £47; donations, £5,796, an increase of £3,188; legacies, however, fell off from £2,174 in 1897 to £692. The festival dinner, presided over by the Duke of Connaught, yielded over £3,500, the largest amount received since 1872. In 1897 the Prince of Wales's Fund granted a donation of £957. In 1898 £1,000 was received from the Fund as an annual subscription. A special gift of £1,000 from an anonymous donor for structural and special repairs was also recorded. During the year 691 patients were treated in the wards—an average of 74 daily. The out-patients numbered 16,158, with an average weekly attendance of 1,218; the total attendances for the year being 63,367. Confidence was expressed that the Ladies' Association, under the presidency of Lady Rothschild, will be attended with conspicuous success. The Chairman referred in terms of warm approval to the help received from the working men's societies, which was especially gratifying as showing that they had the confidence and goodwill of the working people of East London. The committee had also been greatly helped by the secretary, who had worked very hard and most loyally, both in the matter of the reduction of the expenses and in the various methods of raising money. Referring to the movement with regard to the cure of consumption, he wished to recall the fact that their physicians three years ago strongly recommended the open-air system by means of sanatoria, and it was only want of funds which prevented them moving more boldly in the matter. Turning then to the burning question of the inquiry officer, the Chairman reminded the meeting that those who had the management of the hospital had only one desire—to do their best for it. The Hospital Sunday Fund had given them a strong hint to institute inquiries as to the fitness of applicants for help, and they had determined to give the idea a trial. Strictly as an experiment, for three and a half months, which would expire in a few days, they had appointed an inquiry officer so that they would be able to see for themselves if their hospital was being abused. During that time 3,500 different cases had been investigated, and the total number of those rejected as unfit was only about 2 per cent. He admitted the committee did not like the business of inquiry, which was a most invidious one. (Loud and prolonged cheers.) But they had to do their duty to all sections of their subscribers, to those who were really deserving of assistance, and also to the medical men of the neighbourhood, to whom it would be most unjust if they treated those able to pay for treatment.

Mr. Herbert Robertson, M.P., seconded. He reminded his hearers that on Thursday a conference was to be held in conjunction with the working men's societies to settle the question of the inquiry officer, and appealed to the meeting to leave it to that conference. (No! no!)

Sir Edward Sassoon, the treasurer, supported the resolution, but was immediately followed by a number of working-men governors, among whom were Mr. Bartlett, Mr. Smith, and Mr. Parker, who all demanded the deletion of the paragraph in the report which stated that "in accordance with the wishes expressed by the constituents of the Hospital Sunday Fund, an inquiry officer has been appointed."

The Chairman pointed out that this was but a simple statement of fact—the officer had been appointed. He repeated that the arrangement terminated on the 10th inst., and that Thursday's conference would decide whether it should be renewed.

Sir Mancherjee Bhownaggree, M.P., expressed himself in accord with the ideas of the objectors, but suggested, instead of the deletion of the paragraph, the addition to it of words expressive of the pleasure of the committee that only 2 per cent. of the cases had had to be rejected.

Mr. John A. Dickinson, as a working-man member of the committee, appealed to the workers not to press the amendment, which would practically amount to a vote of censure on the committee; and after considerable further discussion it was withdrawn and the report adopted, on the distinct understanding that the meeting was not thereby pledged to countenance, much less to approve of, the idea of inquiry.

The next business was the election of the committee of management, and here again the working men proceeded to assert themselves. Mr. Bartlett proposed the addition to the list of five representatives of working men's societies—a proposition which was promptly seconded and warmly supported.

The Chairman said he thought it would have been more courteous to the committee to have given them notice of a resolution of that kind; and, one word leading to another, the tone of the meeting began to get distinctly dangerous to peace and goodwill. However, in response to various appeals, the number demanded was reduced to four, then to three.

Sir Edward Sassoon, after consultation with his colleagues, said that, taking into account the very strong feeling of the meeting, the committee were prepared to accept two names, and eventually those of Messrs. Pilling and Yetton were, by consent, added to the original motion, which was then carried unanimously.

Thanks to the medical staff were very heartily voted, and other regular business having been concluded, the chairman was about to declare the meeting over, when

Mr. Bartlett again rose to bring up the question of the inquiry officer. They had made up their minds, he declared with emphasis, that the obnoxious questions on the out-patients' letters should be removed, and he wished to learn from the chairman what was to be done in the matter. Once more the question was fiercely discussed, but before long it became apparent that the working men, having had the bit in their teeth throughout the meeting, began to perceive that there was serious danger, if they persisted in taking their own course, of the coach being overturned—in other words, that a split might develop so wide and so deep that the institution, whose welfare they heartily and sincerely desired, could not but suffer. As one of them put it, while feeling that what they demanded was in the best interests of the hospital, they had no wish to run counter to the feelings of the more wealthy individual supporters, and the resolution handed in instructing the committee to call in all letters containing the objectionable inquiries and issue others in their place from which these had been deleted was at length withdrawn on the understanding that, if necessary after Thursday's conference, a special meeting should be called to deal with the subject.

Mr. Bartlett, the fluent and persistent leader of the majority, then proposed a vote of thanks to the chairman, which being carried by acclamation, the meeting terminated.

### THE NEW HOSPITAL FOR WOMEN.

### Opening of the Cancer Ward and Annual Meeting.

On the 7th inst. the Bishop of London presided at the annual meeting of the subscribers to the New Hospital for Women, Euston Road, and afterwards opened the new cancer ward and the Nurses' Home.

The Chairman, in proposing the adoption of the report, recommended brevity in such proceedings, and therefore asked those assembled to take it as read. He remarked that although he could only commend the report in general terms, his own personal satisfaction in doing so was great. The opening of the new buildings began a new period in the history of the institution. He was old enough to remember the time when the idea that a public hospital could be governed by women would have been regarded as impossible. This institution was a proof of how beneficently women could occupy themselves in this way, and with what satisfaction their subscribers regarded their work. The crying want of many hospitals was proper accommodation for the nurses. Their new buildings enabled them to attend to this point. When it was remembered how much the result of their work depend upon efficiency, knowledge, and zeal of the nurses, it was right that a reasonable amount of care should be bestowed upon their personal comfort. Such care put their work on a proper basis, and ensured cheerfulness and contentment amongst them. Through the generosity of a lady benefactor they had been able to give their nurses a home. Through that of another friend they were to-day able to take possession of a cancer ward. There was no malady so painful, none that needed such tender care, as this. They were wise thus to enlarge the borders of their usefulness; the public interest was growing in their work, and their subscription list was increasing. Happily they were free from debt, and were desirous of not incurring it; but as the cost of maintenance must increase with the larger buildings, he trusted that subscribers would come forward and relieve the committee of any anxiety on that score.

Sir Robert Owen seconded the resolution, which was then carried unanimously.

The Rev. H. L. Paget, in proposing the election of the new members of the committee, said that one of the worst bits of Old London had been pulled down to make room for the hospital.

The Rev. James Chadburn (by whose gift of £5,000 the Grace Chadburn ward has been built for five cancer patients) said that if one withheld one's charity until one found a charity without an abuse, one's credit at the bank would not be greatly diminished. For eighteen years he had worked in East London, and had rarely been without a case of cancer on his visiting list. He knew from personal experience what it was to offer vain prayers for a sufferer's release from agony even when all the alleviations of medical skill and wealth were at hand, and bade his hearers picture poverty added to the pain. In the working man's home there was often not even a separate room to be spared for the patient. When one had seen the unselfish devotion of neighbours—poor people who could ill spare the time and money to the sick—one realised that the richest gifts of the wealthy weighed but lightly in the balance. It was with deep joy that he knew from henceforth five beds had been placed at the service of these patients. He regretted that the beds would not be fully endowed, and that a yearly deficit of £75 remained to be covered. He would like to see this deficit apportioned amongst those assembled, and in the event of £50 being forthcoming from them he was prepared to give the other £25.

The report was for nine months only, the hospital having been closed for three months during the extensive alterations and repairs. In that nine months 412 in-patients were treated, and 82 major operations were performed, the death-roll numbering 12. There has been no death in the maternity department, a source of great satisfaction to the staff. Subscriptions amounting to £1,008 have been received; and donations for £1,276. £172 10s. was granted by the Hospital Sunday Fund, and £87 by the Hospital Saturday Fund. The total income was £4,787 4s. 9d.; the excess of payments over that sum 11s. only.

### THE MIDDLESEX HOSPITAL, W.

At the quarterly General Court of Governors, Mr. Henry N. Custance presided. Amongst a large attendance of Governors were the Right Hon. Sir Ralph Thompson, K.C.B., Sir A. T. Watson, Sir Squire Bancroft, Sir Elliott Lees, the Hon. G. W. Spencer Lyttelton, and Dr. W. Cayley.

The report for the year 1898 was read, from which it appeared that the hospital was doing much useful work amongst the poor, 2,996 in and 37,447 out-patients having been treated, whilst 621 had been sent to the hospital's convalescent home. Necessary repairs in the buildings,

&c., and other extraordinary expenditure during the year under review being very heavy, the income was exceeded by £14,495. Sir Ralph Thompson, in moving the adoption of the report, referred to the new department of bacteriology recently established at the hospital, stating that this would prove most useful in research as to the origination of diseases, and especially so with regard to cancer, a great effort being about to be made at the hospital in connexion with the new wing for female cancer patients to thoroughly investigate the causes of and remedies for this disease. Sir Ralph also referred to a proposal now under consideration for the open-air treatment of consumption. In seconding the adoption of the report, Sir Arthur Watson referred to the measures being adopted at the hospital for preventing abuse in the out-patient department, and Mr. Custance, in putting the report to the meeting, also spoke on this subject, stating that as it had been of general interest to the public for the past eighteen months, he thought that it should be made widely known that the Middlesex Hospital had taken effective means to check such abuse. The proceedings closed with a vote of thanks, proposed by Sir Squire Bancroft, to Mr. Custance for presiding.

### CENTRAL LONDON OPHTHALMIC HOSPITAL.

Mr. E. F. Henley, the hon. solicitor, presided on Thursday, 23rd ult., at the annual meeting of the Central London Ophthalmic Hospital, Gray's Inn Road, Mr. Henry Clarke, the late chairman, having been compelled owing to increasing engagements to relinquish that post.

From the report and accounts presented it appeared that the total ordinary income was £1,423, plus £100 in legacies. The total ordinary expenditure was £1,479, the extraordinary expenditure for repairs, &c., amounting to £108. The contributions to the building fund during the year were £698, the balance at credit of this fund being now £2,354. The plans for rebuilding cannot be carried out until at least £5,000 has been obtained, as without this a renewal of the lease will not be granted. It is intended to pull down the two newly-acquired houses adjoining, and rebuild them in a form befitting the modern requirements of an ophthalmic hospital. But as it stands the new annexe—which was opened by the Lady Mayoress—furnishes considerable additional space on the ground floor for the treatment of the out-patients, as well as 15 extra beds, increasing the number available to 28.

In moving the adoption of the report, the Chairman earnestly appealed for assistance in raising the large amount still required for the erection of the new hospital, and to meet the heavy additional expenditure which the enlargements and the increase in the number of patients involve. They were in the midst of a district in which were situated workshops of all descriptions, especially those where accidents to the eyes were frequent; and, in addition to the working men who came to them from these, they had such people as governesses, clerks, and many others who got their living by the use of their eyes.

The adoption of the report having been carried, an alteration of the rules was sanctioned. Formerly it had been the practice, except in the case of the destitute or of those who brought subscribers' letters, to make a charge of half-a-crown a month or twopence an attendance. As altered, the rule provides that admission shall be free to all, but that patients shall be invited to assist the funds by the purchase from the secretary of hospital stamps, as issued by the Prince of Wales's Fund, or otherwise as they can afford. It was stated that since the beginning of the year, when the new plan was put into operation, a great many stamps had thus been disposed of, to the decided benefit of the funds of the hospital.

In returning thanks for a vote of thanks accorded to the medical staff for their services, Mr. Brittin Archer, the senior surgeon, mentioned that they had provided the proper appliances by which the Röntgen Rays might be employed to detect foreign substances in the eye, and expressed his confidence that the sight of many would thereby be saved. He warmly supported the appeal for the building fund, and said that though they were not in monetary debt, and could not on that ground ask for consideration, yet they were morally in debt to the charity for new buildings, which were imperatively necessary for the proper carrying on of the work.

### NATIONAL ORTHOPÆDIC HOSPITAL.

In the absence of the Duke of Marlborough, the president, the chair was taken at the annual general meeting on Tuesday, February 28th, of the National Orthopædic Hospital for the Deformed by Mr. J. R. Cooper, the chairman of the Committee of Management. The report presented expressed the committee's gratification that the close of the year found the hospital in a more favourable financial position than they expected would be the case, a heavy deficit being apparently inevitable until quite late in the year. This was, however, avoided, partly by the receipt of legacies, and partly by timely donations. Though nothing was received from the Prince of Wales's Fund, and a smaller amount from patients' payments, the total receipts were £350 in advance of the previous year, both donations and legacies showing substantial increases. The ordinary income was £2,089, with £400 from legacies, and the ordinary expenditure was £2,446. One hundred and thirty-two children and 80 adults—212 in all—were received as in-patients, while 1,308 out-patients paid 3,240 visits during the year. £175 was raised by the sale of work by the Ladies' Committee as against £132 in 1897. In proposing the adoption of the report and accounts, which he characterised as by far the most satisfactory they had been able to present, the Chairman appealed strongly for help to carry out the rebuilding of the Bolsover Street portion of the hospital. There was great need for the increased accommodation which would thereby be provided. They were always full, and at the present time there were 40 cases waiting for admission. These were nearly all children, and it was obvious that much harm might be done by delay. It was a great point to deal with such cases promptly, so that the malformation might not become set; if they were not dealt with it might entail on the child a lifetime of sorrow and suffering. The report was adopted, and votes of thanks to the committees and to the officers and staff brought the proceedings to a close.

### ST. MARY'S CHILDREN'S HOSPITAL, PLAISTOW.

The annual Court of the Governors of this hospital was held on Thursday, the 16th ult., the Venerable the Archdeacon of Essex in the chair. The report of the Committee of Management showed a large increase in the work of the hospital, the numbers for the past three years being: in 1896, 198; in 1898, 661 in-patients; and in 1896, 3,660; in 1898, 10,094 out-patients. The number of beds has been increased from 30 to 38, and the average number occupied all the year round was 35. Of the cases treated by far the greater number, amounting to 50 per cent., have been among the families of labourers, of whom 15 per cent. were classified as dock labourers. Among the other employments specified are bricklayers, painters, carpenters, gas workers, railway men, workers in various factories, &c., while a large number of the patients have been widows and orphans. During the year an ophthalmic department has been started, as there is no special hospital, nor a general hospital with an ophthalmic department, anywhere in the neighbourhood. This new branch of the work has already been of great benefit to many of the poor. The receipts of the hospital amounted to £2,616 9s. 5d., and the expenditure to £2,772 14s. 1d., an increase of £700 over that of the previous year, and caused entirely by the much larger number of patients treated. Including a debt to the building fund, the hospital is now £500 in debt, and the committee are asking for contributions to clear this off, and to enable them to maintain the work. An enlargement is urgently needed, as the building is totally inadequate for the work now carried on, but owing to want of funds, it has had to be postponed. The report having been adopted, representatives of the governors were elected as members of the Committee of Management for the ensuing year.

